# Socio-demographic factors and psychological distress in Indigenous and non-Indigenous Australian adults aged 18-64 years: analysis of national survey data

**DOI:** 10.1186/1471-2458-12-95

**Published:** 2012-02-01

**Authors:** Joan Cunningham, Yin C Paradies

**Affiliations:** 1 Menzies School of Health Research, Charles Darwin University, PO Box 41096, Casuarina, Darwin, NT 0811, Australia; 2 McCaughey Centre, Melbourne School of Population Health, University of Melbourne, Level 5, 207 Bouverie St, Melbourne, VIC 3010, Australia

## Abstract

**Background:**

Indigenous Australians are known to be at greater risk of morbidity and mortality from mental health related conditions, but most available data relate to the use of mental health services, and little is known about other aspects of social and emotional wellbeing. Using the first available nationally representative data, we examined the prevalence and patterning of psychological distress among Indigenous Australian adults and compared these with corresponding data from the non-Indigenous population.

**Methods:**

The analysis used weighted data on psychological distress, as measured by a modified Kessler Psychological Distress score (K5), and a range of socio-demographic measures for 5,417 Indigenous and 15,432 non-Indigenous adults aged 18-64 years from two nationally representative surveys. Very high psychological distress (VHPD) was defined as a K5 score ≥ 15 (possible range = 5-25).

**Results:**

Indigenous adults were about three times more likely than non-Indigenous adults to be classified with VHPD: 14.5% (95% confidence interval (CI) 12.9-16.0%) versus 5.5% (95% CI 5.0-5.9%). After adjusting for age, most socio-demographic variables were significantly associated with VHPD in both populations, although the relative odds were generally larger among non-Indigenous people. Indigenous people in remote areas had a lower prevalence of VHPD than their non-remote counterparts, and only marital status, main language, and food insecurity were significantly associated with VHPD in remote areas.

**Conclusions:**

Higher absolute levels of VHPD combined with smaller socio-demographic gradients in the Indigenous population suggest the importance of risk factors such as interpersonal racism, marginalization and dispossession, chronic stress and exposure to violence that are experienced by Indigenous Australians with common and/or cross-cutting effects *across *the socioeconomic spectrum. The lower prevalence of VHPD and lack of association with many socio-demographic variables in remote areas suggests either that the instrument may be less valid for Indigenous people living in remote areas or that living in an Indigenous majority environment (such as exists in most remote communities) may mitigate the risk of psychological distress to some degree.

## Background

Indigenous Australians (Aboriginal and Torres Strait Islander people), who represent approximately 2.5% of the total Australian population, are disadvantaged relative to other Australians across a broad range of social and health indicators and are at greater risk of morbidity and mortality from mental health-related conditions [1]. For example, in 2005-06, Indigenous Australians were 2-3 times more likely than other Australians to be admitted to hospital for intentional self-harm and 1.9 times more likely to be admitted for mental and behavioural disorders [1]. Indigenous Australians are also more likely to be exposed to a range of life stressors. In 2002, Indigenous people were about twice as likely as non-Indigenous Australians to report the death of a family member/close friend in the previous year and about 3.5 times more likely to report being affected by someone's alcohol/drug problems or abuse/violent crime [1]. Mental disorders accounted for an estimated 15.5% of the total burden of disease and injury (as measured by Disability Adjusted Life Years) among Indigenous Australians in 2003, second only to cardiovascular disease (17.5%) [1].

In keeping with Indigenous understandings of health [2], the emphasis in Australia in recent years has been on the broader concept of social and emotional wellbeing, rather than the more narrow--and arguably more negative--concept of mental ill-health. However, progress has been limited by the paucity of data, with most available information to date relating to the use of health services for mental illness. Social and emotional wellbeing is one of nine key result areas in the current National Strategic Framework for Aboriginal and Torres Strait Islander Health, and one of the nominated actions for governments is to improve the evidence base about social and emotional wellbeing in culturally appropriate and sensitive ways [3].

The collection of data on the social and emotional wellbeing of Indigenous Australians has long been a contested and sensitive area, with slow progress over many years. Following extensive consultation (including a stakeholder workshop in 2003), negotiation and field testing, an "interim" social and emotional wellbeing module was developed through a partnership involving the Australian Bureau of Statistics (ABS), the Australian Institute of Health and Welfare and the National Aboriginal Community Controlled Health Organisation [4]. This interim module was first used in the ABS's 2004-05 National Aboriginal and Torres Strait Islander Health Survey (NATSIHS). It included eight domains: 1) psychological distress; 2) impact of psychological distress; 3) life stressors; 4) discrimination; 5) anger; 6) removal from natural family; 7) cultural identification; and 8) positive wellbeing. Although it was recognised that other domains were of interest, it was agreed by the key stakeholders that no satisfactory measures were available at that time [4]. For most of the measures included in the interim module, there were no comparable data for the non-Indigenous population. One exception was psychological distress, for which data for the non-Indigenous population were available from the ABS's 2004-05 National Health Survey (NHS).

Some basic data on psychological distress from the NATSIHS have been presented previously [1,4,5], but more detailed analysis is required to adequately understand the socio-demographic patterns of psychological distress within the Indigenous population, as well as to assess the utility and robustness of the measure. In other populations, low socioeconomic status (SES) has been associated with psychological distress [6-8], but it is not known whether, or to what extent, this holds true in the Indigenous population. Traditional measures of SES are not necessarily equivalent across social groups, and they may not adequately measure all relevant aspects of what they purport to measure [9,10]. Although exposure to interpersonal racism occurs across the SES spectrum, increased self-reported interpersonal racism has been associated with high SES, at least in some settings [11-13]. Such exposure could potentially reduce the SES gradient in psychological distress. In addition, there are recognised differences in the cultural identity and SES of Indigenous Australians living in remote and non-remote areas [14], and these may impact on the size and nature of the gradient for people living in different areas.

The aims of the current study are: 1) to present more detailed data on psychological distress among Indigenous Australians; 2) to examine the relationships between socio-demographic measures and an indicator of very high psychological distress (VHPD) among a nationally representative sample of Indigenous Australian adults; and 3) to compare these relationships with corresponding patterns in the non-Indigenous population.

## Methods

Data for Indigenous and non-Indigenous adults aged 18-64 years were taken from the NATSIHS and the NHS, two national surveys conducted in parallel by the ABS in 2004-05. These two surveys had very similar content and in most cases the wording of questions on particular topics was identical [15]. This analysis is limited to responses to questions deemed by the ABS to be comparable across the two surveys [16].

Extensive details on survey methodology have been published elsewhere [5,15-19]. Briefly, both surveys were conducted using multi-stage sampling strategies; the first stage involved random selection of either communities or census collection districts (CD), and subsequent stages involved selection of dwellings and individuals within households [17,19]. Both the NHS and NATSIHS samples were designed to provide reliable estimates for Australia as a whole as well as for selected sub-national areas, such as State/Territory, capital city versus balance of state within each state (NHS) and remote versus non-remote areas (NATSIHS). Indigenous respondents from the NHS were included with NATSIHS data to provide Indigenous population estimates [17]. Both surveys were limited to usual residents of private dwellings and conducted by trained ABS interviewers. Very remote areas were out of scope in the NHS but in scope for the NATSIHS. In the NHS and in non-remote areas in the NATSIHS, data were collected using Computer Assisted Interviews. In remote areas of the NATSIHS, pen and paper interview forms were used and some questions were simplified or deleted. After accounting for sample loss (e.g. dwellings out of scope or vacant, households with no adults, etc.), 89% of selected households in the NHS were classified as fully/adequately responding [19]. Corresponding figures in the NATSIHS were 85.5% in remote Indigenous communities and 83.4% in other areas [17]. More details about the design, conduct and results of the surveys are available elsewhere [5,15-19].

To allow data access to interested researchers, the ABS created a Confidentialised Unit Record File (CURF) for the NATSIHS. This file includes unit records for Indigenous respondents of the 2004-05 NATSIHS and the 2004-05 NHS, as well as unit records for non-Indigenous respondents from the 2004-05 NHS [16]. Although the CURF contains unit records for participants of all ages, this analysis is limited to data from the 20,849 adult respondents (5,417 Indigenous and 15,432 non-Indigenous) aged 18-64 years. Children less than 18 years were not asked questions about psychological distress. The exclusion of those aged ≥ 65 years was due to uncertainty about the applicability of socioeconomic indicators among older people, as well as the relatively small size of this group in the Indigenous population [1].

### Definition of psychological distress

Psychological distress was assessed in the NATSIHS using a modified version of the Kessler 10 Psychological Distress Scale (K10) [20]. The K10 scale was designed to measure non-specific psychological distress in the depression/anxiety spectrum and consists of ten questions [20]. The K10 and a shorter, six-question subset, known as the K6, have been shown to be robust, with the K6 performing virtually as well as the K10, and both significantly out-performing other instruments [20-22].

The instrument used in the current study (hereafter referred to as the K5) includes five of the questions included in the K10, or all but one of the six questions included in the K6 [4]. Concerns were raised by stakeholders that the K6 question about feeling worthless might be considered offensive by some Indigenous respondents [4]. Based on a range of factors, including the perceived robustness of the K10 and K6, the results of analysis of data from the New South Wales Health Survey, support from state/territory health authorities and consultation with Kessler himself, this question was dropped and the remaining five questions were retained [4,17]. Slight wording changes were made to two questions: 'hopeless' was replaced by 'without hope' and 'restless or fidgety' was replaced by 'restless or jumpy' [4]. In addition, it appears that ABS surveys have consistently used the wording 'so sad that nothing could cheer you up' in place of 'so depressed that nothing could cheer you up' [23,24], and this wording was retained in the K5 [4].

Thus the K5 included the following questions: During the past four weeks, about how often did you feel...? a) nervous; b) without hope; c) restless or jumpy; d) so sad that nothing could cheer you up; e) that everything was an effort. Response options for each of the five questions were: none of the time (1); a little of the time (2); some of the time (3); most of the time (4); all of the time (5). Responses were summed up over the five questions to produce a possible range of 5-25, with higher scores indicating greater psychological distress. This is consistent with previous Australian scoring of the K10 [23,24], but is slightly different to the scoring system used elsewhere, in which scores on each question range from 0 to 4, rather than 1 to 5. The complete K10 was administered in the NHS, but only the five questions corresponding to the K5 were included for NHS participants in the NATSIHS CURF.

Due to the potentially sensitive nature of the questions, 'refusal' was available as a response option to NATSIHS participants on all five questions. Participants who selected this option were coded as missing. The number of participants with missing responses on individual questions ranged from 56 (1.0%) to 80 (1.5%) among Indigenous respondents and from 16 (0.1%) to 19 (0.1%) among non-Indigenous respondents. A total of 117 Indigenous respondents (2.2%) and 21 non-Indigenous respondents (0.1%) were missing on at least one of the five questions; they have been excluded from the analysis, as a K5 score could not be calculated for them.

For those not functionally literate in English, questions were translated into the respondent's main language by an Indigenous facilitator, and responses were given to the interviewer in English [4]. Referrals were made as necessary either to a local health clinic (in remote areas) or to the nearest Aboriginal Medical Service (in non-remote areas) [4].

For the purposes of this analysis, the following categories of psychological distress were defined, based on K5 score ranges: low = 5-7.9; moderate = 8-11.9; high = 12-14.9; very high = 15-25. These ranges are consistent with initial NATSIHS survey output published by the Australian Institute of Health and Welfare [4] and the ABS [1,5].

Additional data on the impact of psychological distress were available for Indigenous participants only, including: whether the respondent had days in which they were unable to work or carry out normal activities due to their feelings in the last four weeks; whether the respondent saw a doctor or other health professional about their feelings in the last four weeks; and how often physical health problems were the main cause of feelings in the last four weeks. No comparable data were available for non-Indigenous participants in the NATSIHS CURF.

### Socio-demographic factors

Information was available on a range of socioeconomic and demographic factors, as shown in Table 1. Information about age and sex of household members, marital status, and whether the respondent was currently attending school was provided by 'any responsible adult' within the household; information about the dwelling and the income of non-participant household members (required to calculate household income) was provided by a household 'spokesperson', chosen on the basis of his or her ability to provide accurate information. Information relating to geography (including remoteness classification and area-level disadvantage score) was provided by the ABS based on the CD in which the selected dwelling was located. All other information used in this analysis was provided by the respondent [17].

**Table 1:** Socio-demographic characteristics of Indigenous and non-Indigenous Australians aged 18-64 years, 2004-05*

	Indigenous			Non-Indigenous
		
	Remote	Non-remote	Total	
	% (95% CI)†	% (95% CI)†	% (95% CI)†	% (95% CI)†
Age (years)				
18-24	20.8 (18.0-23.6)	23.9 (22.3-25.5)	23.1 (21.7-24.4)	15.1 (14.8-15.4)
25-34	29.2 (27.1-31.4)	28.0 (27.0-29.0)	28.4 (27.7-29.0)	22.4 (22.3-22.6)
35-44	27.0 (24.6-29.4)	22.8 (21.7-24.0)	24.0 (23.5-24.5)	23.5 (23.4-23.7)
45-54	15.6 (13.9-17.3)	16.3 (15.5-17.1)	16.1 (15.7-16.4)	22.0 (21.8-22.1)
55-64	7.4 (6.1-8.6)	8.9 (7.0-10.9)	8.5 (7.1-9.9)	17.0 (16.9-17.1)
Sex				
Male	46.7 (44.1-49.2)	46.8 (45.3-48.3)	46.8 (45.6-47.9)	49.8 (49.6-50.1)
Female	53.3 (50.8-55.9)	53.2 (51.7-54.7)	53.2 (52.1-54.4)	50.2 (49.9-50.4)
Marital status‡				
Married	48.6 (44.1-53.1)	27.1 (24.4-29.8)	33.1 (30.8-35.4)	58.7 (57.6-59.8)
Not married	51.4 (46.9-55.9)	72.9 (70.2-75.6)	66.9 (64.6-69.2)	41.3 (40.2-42.4)
Main language spoken at home				
English	52.0 (46.8-57.1)	99.1 (98.7-99.6)	86.0 (84.5-87.5)	90.8 (89.9-91.7)
Not English	48.0 (42.9-53.2)	0.9 (0.4-1.3)	14.0 (12.5-15.5)	9.2 (8.3-10.1)
Highest year of school completed				
Year 12	14.9 (11.8-17.9)	26.8 (23.9-29.8)	23.5 (21.2-25.8)	52.5 (51.2-53.8)
Year 11	14.8 (12.4-17.2)	12.4 (10.7-14.0)	13.0 (11.7-14.4)	10.9 (10.3-11.6)
Year 10	31.2 (28.2-34.2)	31.2 (28.9-33.6)	31.2 (29.4-33.1)	24.7 (23.7-25.7)
Year 9	13.1 (10.9-15.3)	14.2 (12.5-15.9)	13.9 (12.5-15.3)	6.3 (5.8-6.7)
≤ Year 8§	26.0 (22.8-29.3)	15.4 (13.6-17.1)	18.3 (16.7-20.0)	5.6 (5.1-6.1)
Highest non-school qualification				
Post-graduate degree	0.7 (0.3-1.1)	2.3 (1.2-3.4)	1.9 (1.0-2.7)	6.2 (5.8-6.7)
Bachelor's degree	1.4 (0.8-2.0)	3.5 (2.6-4.4)	2.9 (2.3-3.6)	14.5 (13.8-15.3)
Diploma	2.4 (1.5-3.2)	5.6 (4.2-7.0)	4.7 (3.7-5.7)	9.7 (9.1-10.3)
Certificate	19.3 (16.4-22.1)	26.0 (23.6-28.4)	24.2 (22.2-26.1)	26.0 (25.0-27.1)
No qualifications	76.3 (73.2-79.4)	62.6 (59.7-65.4)	66.4 (64.1-68.6)	43.5 (42.5-44.5)
Employment status				
Employed	55.3 (51.5-59.1)	54.4 (51.3-57.5)	54.7 (52.2-57.1)	76.1 (75.3-76.8)
Unemployed	7.1 (5.5-8.6)	8.4 (7.0-9.9)	8.1 (6.9-9.2)	3.0 (2.7-3.4)
Not in the labour force	37.7 (34.1-41.2)	37.1 (34.3-40.0)	37.3 (35.0-39.6)	20.9 (20.1-21.7)
Housing tenure				
Owner/purchaser‖	6.8 (4.1-9.5)	31.6 (28.1-35.1)	24.7 (22.1-27.3)	n/a**
Renter/other tenure	93.2 (90.5-95.9)	68.4 (64.9-71.9)	75.3 (72.7-77.9)	n/a**
Reported food insecurity††				
Yes	36.5 (33.0-40.1)	20.1 (17.7-22.4)	24.6 (22.7-26.6)	5.6 (5.1-6.0)
No	63.5 (59.9-67.0)	79.9 (77.6-82.3)	75.4 (73.4-77.3)	94.4 (94.0-94.9)
Equivalised household income quintile‡‡				
1 (lowest)	37.1 (32.8-41.5)	32.4 (29.6-35.1)	33.7 (31.4-36.1)	11.3 (10.7-11.9)
2	25.9 (22.4-29.4)	20.0 (17.6-22.4)	21.6 (19.7-23.6)	13.1 (12.5-13.8)
3	8.7 (6.7-10.8)	16.4 (13.9-18.9)	14.3 (12.4-16.1)	16.9 (16.1-17.6)
4	5.5 (2.7-8.3)	11.0 (8.8-13.1)	9.4 (7.7-11.2)	19.5 (18.7-20.2)
5 (highest)	3.2 (1.6-4.8)	6.0 (4.4-7.6)	5.2 (4.0-6.4)	21.7 (20.7-22.7)
Not known or not stated	19.4 (14.9-23.9)	14.1 (11.9-16.3)	15.6 (13.6-17.6)	17.5 (16.6-18.4)
SEIFA quintile§§				
1 (most disadvantaged)	72.5 (62.6-82.4)	41.8 (35.1-48.4)	49.3 (43.7-55.0)	17.1 (15.7-18.5)
2	11.5 (5.3-17.8)	21.8 (16.9-26.6)	19.3 (15.2-23.3)	19.0 (17.4-20.7)
3	11.3 (3.7-18.9)	20.8 (15.8-25.8)	18.5 (14.3-22.7)	20.3 (18.4-22.2)
4	4.6 (0.0-9.5)	10.4 (7.5-13.4)	9.0 (6.4-11.6)	21.3 (19.5-23.0)
5 (least disadvantaged)	0.1 (0.0-0.3)	5.2 (2.9-7.5)	3.9 (2.2-5.7)	22.3 (20.0-24.7)
Area of residence‖‖				
Non-remote	---	100.0	72.2 (70.6-73.7)	100.0
Remote	100.0	---	27.8 (26.3-29.4)	---***

* Source: Weighted data from the National Aboriginal and Torres Strait Islander Health Survey 2004-05 confidentialised unit record file (CURF) [16]. Based on data from 5,417 Indigenous participants (2,197 remote and 3,220 non-remote) and 15,432 non-Indigenous participants

† CI, confidence interval. Proportions are weighted to provide population estimates. Totals are based on those with non-missing data, except for equivalised household income, for which a separate unknown category is shown

‡ Respondents who were reported to be living in a 'couple relationship' (including same sex relationships) were classified as married. Both registered marriages and defacto relationships were included, as long as both partners lived in the same household. Respondents who did not report a couple relationship, as well as those whose partner did not usually live in the same household, were classified as not married [17]

§ Includes those who never went to school

‖ Includes ownership/purchase by any of the dwelling's occupants (not necessarily the respondent) [5,17]

** n/a, not available

†† Food insecurity was based on the response to a question about whether, in the past 12 months, the respondent had run out of food and couldn't afford to buy more [17]

‡‡ Gross weekly equivalised cash income of household, which takes into account household size and composition, was estimated using the OECD scale [5,17]. Quintiles were determined based on all-Australian figures. That is, the same category boundaries were used for both Indigenous and non-Indigenous participants

§§ SEIFA, Socioeconomic Index for Areas, Index of Relative Disadvantage. SEIFA quintile was based on the 2001 score for the CD of the selected dwelling and is used as a measure of area-level disadvantage [5,17]. Quintiles were determined based on all-Australian figures. That is, the same category boundaries were used for both Indigenous and non-Indigenous respondents

‖‖ Classified according to the Australian Standard Geographical Classification remoteness classification (based on the ARIA + index) [5,17]

*** Out of scope

Respondents who were reported as living in a 'couple relationship' (including same sex relationships) were classified as married. Both registered marriages and defacto relationships were included, as long as both partners lived in the same household. Respondents who did not report a couple relationship, as well as those whose partner did not usually live in the same household, were classified as not married.

The main language spoken at home was collected for adults aged 18 years and over, based on self-report. The NATSIHS CURF included three categories for main language: English only; Australian Indigenous languages; and other languages. For the purposes of this analysis, the last two categories were combined and main language was classified as English or not English. Main language was included in the analysis because the outcome measure was based on self-report, which could conceivably be influenced by facility with English and/or translation issues.

Those reported as still at school (n = 67) were not asked about educational attainment or non-school qualifications. They have been coded as missing on both variables. Those whose educational attainment was not stated (n = 2) and those whose level of qualifications could not be determined (n = 222) were coded as missing on this variable.

Gross weekly household equivalised income, which takes into account household size and composition, was estimated using the Organisation for Economic Co-operation and Development (OECD) scale [17]. Quintiles were determined based on all-Australian figures. That is, the same categories were used for both Indigenous and non-Indigenous participants.

Home ownership was only available in the CURF for Indigenous respondents (missing for n = 41), and was based on whether the home was owned or being purchased by any of its occupants (not necessarily the respondent) [5,17].

Food insecurity was based on the response to a question about whether, in the past 12 months, the respondent had run out of food and couldn't afford to buy more.

Area of residence was classified according to the Australian Standard Geographical Classification remoteness classification (based on the ARIA + index) into major cities, inner regional, outer regional, and remote/very remote [5]. However, only two categories (remote and non-remote) were available in the CURF. ABS documentation indicates that the remote category was to be used only for Indigenous respondents [16]; area of residence was therefore re-coded to missing for 312 non-Indigenous respondents whose residence was categorised as remote.

Area-level disadvantage was based on the 2001 Socioeconomic Indexes for Areas (SEIFA) Index of Disadvantage score for the CD of the selected dwelling [19]. Quintiles were determined based on all-Australian figures. That is, the same categories were used for both Indigenous and non-Indigenous respondents. Those with SEIFA quintile not known (n = 313) were coded as missing.

### Statistical analysis

All analyses were conducted using Stata version 10.0 (StataCorp LP, College Station, TX) via the ABS's Remote Access Data Laboratory (RADL). Under the RADL system, analysts submit statistical code to the ABS; the code is then run and the output made available to the analyst through a password-protected web account. Analysts do not have direct access to unit record data at any time, and there are limits placed on the commands and outputs that are allowed, in order to protect the security and confidentiality of the data [25].

All analyses used ABS-generated person-weights (or expansion factors) to adjust for disproportionate sampling of some groups. The estimates produced in this manner apply to the population as a whole, and not just the sample [16,26]. Standard errors and 95% confidence intervals (CI) were calculated using replicate weights produced by the ABS using the Jackknife method (250 replicate weights for Indigenous respondents, 60 for non-Indigenous respondents) [16,26]. These replicate weights allow estimation of standard errors taking into account the complex design and weighting procedures used in the surveys [17,26]. Because of the different number of replicate weights in the two groups, separate analysis was required to estimate standard errors for the Indigenous and non-Indigenous groups. Although Stata version 10 incorporates a suite of procedures to analyse complex survey data, these commands cannot be utilised in the RADL system (Therese Lalor, ABS, personal communication, May 2009). Instead, commands from the *svr *module written by Nick Winter [27] were used.

Logistic regression was conducted separately for Indigenous and non-Indigenous groups due to the different numbers of replicate weights for the two groups. Preliminary analysis suggested the presence of interactions between sex and other variables, with *p*-values < 0.05 in the Indigenous group and/or the non-Indigenous group for interaction terms involving sex and marital status, having a university degree or a certificate level qualification, being out of the labour force or unemployed, and equivalised household income quintile. As a result, all analyses were performed separately for males and females. All models were adjusted for age group (18-24, 25-34, 35-44, 45-54 and 55-64 years), with socioeconomic variables assessed individually. Participants with missing data were excluded only from analyses involving the variable for which they were missing data. The proportion of participants with missing data was small for all variables with the exception of equivalised household income quintile, which was not available for 2,941 respondents (14.1% overall, including 14.4% of Indigenous respondents and 14.0% of non-Indigenous respondents). Analyses were conducted with these respondents coded as missing, as well as with them included using a special category of household income unknown. Where possible, separate models are presented for Indigenous people in remote and non-remote areas. However, for some variables (most notably SEIFA score and main language) there was relatively little variation within one of the remoteness categories (Table 1).

### Ethics approval

This study was approved by both the Aboriginal sub-committee and the main committee of the Human Research Ethics Committee of the Northern Territory Department of Health and Families and Menzies School of Health Research.

## Results

The socio-demographic profile of the Indigenous population was significantly different from that of the non-Indigenous population, with a younger age distribution, lower educational attainment, and greater levels of disadvantage across a range of indicators (Table 1). There were also marked differences within the Indigenous population according to remoteness of residence.

### Prevalence of very high psychological distress (VHPD)

Over 1 in 7 Indigenous people (14.5%, 95% CI 12.9-16.0) aged 18-64 were classified as having VHPD (Table 2). This figure was almost three times that observed in the non-Indigenous population (5.5%, 95% CI 5.0-5.9). In both the Indigenous and non-Indigenous populations, females were more likely than males to be classified as having VHPD. Among Indigenous people, the gender gap was greater in remote areas (16.2% of females, 8.0% of males with VHPD) than non-remote areas (18.2% of females, 11.9% of males with VHPD) (Table 2).

**Table 2:** Level of psychological distress by sex, remoteness and Indigenous status, Australian adults aged 18-64 years, 2004-05*

	Psychological distress category†‡			
	
	Low	Moderate	High	Very high
	% (95% CI§)	% (95% CI§)	% (95% CI§)	% (95% CI§)
Indigenous males				
Non-remote	43.3 (38.9-47.6)	33.7 (30.4-37.1)	11.2 (8.6-13.8)	11.9 (9.1-14.6)
Remote	53.0 (46.0-60.0)	29.2 (23.6-34.8)	9.8 (6.7-12.8)	8.0 (5.2-10.9)
Total	45.9 (42.2-49.6)	32.5 (29.6-35.4)	10.8 (8.7-12.8)	10.8 (8.7-13.0)
Indigenous females				
Non-remote	31.0 (27.6-34.4)	36.5 (32.8-40.2)	14.3 (11.9-16.8)	18.2 (15.6-20.7)
Remote	36.8 (32.2-41.5)	28.5 (25.0-31.9)	18.5 (15.3-21.7)	16.2 (12.9-19.5)
Total	32.6 (29.9-35.2)	34.3 (31.5-37.2)	15.4 (13.5-17.4)	17.6 (15.6-19.7)
Indigenous persons				
Non-remote	36.7 (33.9-39.6)	35.2 (32.8-37.6)	12.8 (11.0-14.7)	15.2 (13.2-17.2)
Remote	44.4 (40.0-48.9)	28.8 (25.8-31.8)	14.4 (12.1-16.6)	12.4 (10.2-14.5)
Total	38.8 (36.5-41.1)	33.5 (31.5-35.4)	13.3 (11.8-14.8)	14.5 (12.9-16.0)
Non-Indigenous				
Males	55.7 (54.3-57.2)	33.3 (31.9-34.6)	6.5 (5.7-7.3)	4.5 (3.9-5.1)
Females	49.9 (48.4-51.3)	34.2 (32.8-35.7)	9.4 (8.7-10.2)	6.5 (5.8-7.1)
Persons	52.8 (51.7-53.8)	33.8 (32.7-34.8)	8.0 (7.5-8.5)	5.5 (5.0-5.9)

* Source: Weighted data from the National Aboriginal and Torres Strait Islander Health Survey 2004-05 confidentialised unit record file (CURF) [16]

† Categories are based on K5 score ranges as follows: low = 5-7.9; moderate = 8-11.9; high = 12-14.9; very high = 15-25

‡ Excludes those with missing values. The proportion of participants with missing responses on individual questions ranged from 1.0% to 1.5% among Indigenous participants and 0.10%-0.12% among non-Indigenous respondents; a total of 2.2% of Indigenous respondents and 0.1% of non-Indigenous respondents were missing a response to at least one of the five questions comprising the K5

§ CI, confidence interval. Proportions are weighted to provide population estimates

### Association of VHPD with SES indicators

**Figure 1:**
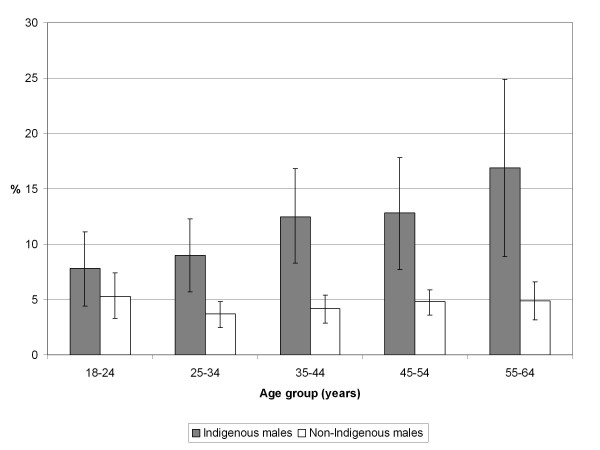
**Prevalence (% and 95% confidence interval) of very high psychological distress in Australian adult males by age and Indigenous status, 2004-05**. Source: Weighted data from the National Aboriginal and Torres Strait Islander Health Survey 2004-05 confidentialised unit record file [16].

**Figure 2:**
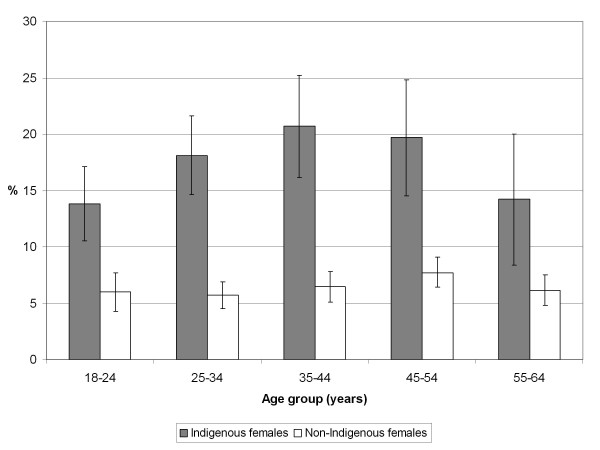
**Prevalence (% and 95% confidence interval) of very high psychological distress in Australian adult females by age and Indigenous status, 2004-05**. Source: Weighted data from the National Aboriginal and Torres Strait Islander Health Survey 2004-05 confidentialised unit record file [16].

VHPD was more commonly reported for Indigenous than non-Indigenous people in every age group (Figures 1 and 2). After adjusting for age, most of the independent variables examined were significantly associated with VHPD among non-Indigenous males and females and among Indigenous males and females in non-remote areas, although the relative odds were generally larger for non-Indigenous males and females than for their Indigenous counterparts (Tables 3 and 4).

**Table 3:** Age-adjusted relative odds of very high psychological distress by socioeconomic status variables for Indigenous and non-Indigenous Australian males aged 18-64 years, 2004-05*

	Remote Indigenous males	Non-remote Indigenous males	All Indigenous males	Non-Indigenous males
	OR (95% CI)†	OR (95% CI)†	OR (95% CI)†	OR (95% CI)†
Marital status				
Married	**0.4 (0.2-0.9)**	1.1 (0.6-1.8)	0.8 (0.5-1.2)	**0.4 (0.3-0.6)**
Not married	1.0	1.0	1.0	1.0
Main language				
English	1.0	---	1.0	1.0
Not English	**0.4 (0.2-0.9)**	---	**0.4 (0.2-0.8)**	1.0 (0.6-1.7)
Highest year of school completed				
Year 10 or more	1.0	1.0	1.0	1.0
Less than Year 10‡	0.7 (0.3-1.5)	**2.7 (1.7-4.3)**	**1.9 (1.3-2.8)**	**3.2 (2.3-4.6)**
Highest non-school qualification				
Bachelor/post-graduate degree	---	**0.1 (0.0-0.5)**	**0.1 (0.0-0.6)**	**0.2 (0.1-0.4)**
Diploma	---	**0.1 (0.0-0.7)**	**0.2 (0.0-0.8)**	**0.5 (0.3-1.0)**
Certificate	1.0 (0.4-2.8)	0.6 (0.3-1.1)	0.7 (0.4-1.2)	**0.6 (0.4-0.8)**
No qualifications	1.0	1.0	1.0	1.0
Employment status				
Employed	1.0	1.0	1.0	1.0
Unemployed	2.6 (0.8-8.6)	**5.0 (2.4-10.4)**	**4.3 (2.3-7.9)**	**7.2 (4.1-12.7)**
Not in the labour force	1.7 (0.7-3.8)	**5.0 (2.9-8.8)**	**3.8 (2.4-6.0)**	**7.9 (5.5-11.3)**
Housing tenure				
Owner/purchaser	0.8 (0.1-4.5)	**0.4 (0.2-0.7)**	**0.5 (0.3-0.8)**	---
Renter/other tenure	1.0	1.0	1.0	---
Ran out of food and couldn't afford to buy more (last 12 months)				
Yes	**2.3 (1.1-4.7)**	**4.1 (2.5-6.7)**	**3.1 (2.0-4.6)**	**6.2 (4.2-9.1)**
No	1.0	1.0	1.0	1.0
Equivalised household income quintile§				
1 (lowest)	1.5 (0.2-11.5)	3.9 (0.7-22.0)	3.0 (0.9-10.5)	**18.9 (10.4-34.2)**
2	0.7 (0.1-5.2)	3.0 (0.4-20.4)	2.0 (0.5-8.3)	**8.6 (4.5-16.4)**
3	0.6 (0.0-10.8)	1.0 (0.1-7.2)	0.9 (0.2-4.0)	**3.8 (2.1-7.2)**
4	---	1.0 (0.1-8.0)	0.8 (0.1-4.0)	2.1 (1.0-4.3)
5 (highest)	1.0	1.0	1.0	1.0
Not known/not stated	1.2 (0.2-9.6)	2.2 (0.3-14.4)	1.8 (0.5-7.1)	**5.2 (2.9-9.3)**
	Trend‖: *p *= 0.16	Trend‖: ***p *< 0.001**	Trend‖: ***p *< 0.001**	Trend‖: ***p *< 0.001**
SEIFA quintile**				
1 (most disadvantaged)	---	1.3 (0.3-5.2)	1.0 (0.3-3.9)	**7.1 (4.0-12.5)**
2	---	0.7 (0.1-3.1)	0.7 (0.2-3.2)	**4.6 (2.4-8.7)**
3	---	0.4 (0.1-1.9)	0.4 (0.1-1.8)	**4.3 (2.3-7.8)**
4	---	0.8 (0.2-2.7)	0.7 (0.2-2.4)	**4.4 (2.4-8.0)**
5 (least disadvantaged)	---	1.0	1.0	1.0
	Trend: n/a	Trend: *p *= 0.16	Trend: *p *= 0.24	Trend: ***p *< 0.001**

* Source: Weighted data from the National Aboriginal and Torres Strait Islander Health Survey 2004-05 confidentialised unit record file (CURF) [16]. Very high psychological distress is defined as a K5 score of ≥ 15

† OR, odds ratio; CI, confidence interval. All estimates are adjusted for age group (18-24, 25-34, 35-44, 45-54 and 55-64 years). Figures in bold are statistically significant at *p *< 0.05

‡ Includes those who never went to school

§ Gross weekly equivalised cash income of household, using the OECD scale [17]. Quintiles are based on national figures

‖ Trend analysis excludes those with equivalised household income quintile 'not known/not stated'

** SEIFA, Socioeconomic Index for Areas, Index of Relative Disadvantage. Quintiles are based on national figures

**Table 4:** Age-adjusted relative odds of very high psychological distress by socioeconomic status variables for Indigenous and non-Indigenous Australian females aged 18-64 years, 2004-05*

	Remote Indigenous females	Non-remote Indigenous females	All Indigenous females	Non-Indigenous females
	OR (95% CI)†	OR (95% CI)†	OR (95% CI)†	OR (95% CI)†
Marital status				
Married	**0.4 (0.3-0.7)**	**0.5 (0.3-0.7)**	**0.5 (0.3-0.6)**	**0.5 (0.4-0.6)**
Not married	1.0	1.0	1.0	1.0
Main language				
English	1.0	1.0	1.0	1.0
Not English	**0.4 (0.3-0.7)**	2.4 (0.7-8.9)	**0.6 (0.4-0.9)**	**1.6 (1.1-2.3)**
Highest year of school completed				
Year 10 or more	1.0	1.0	1.0	1.0
Less than Year 10‡	1.1 (0.7-1.7)	**2.2 (1.5-3.2)**	**1.8 (1.3-2.4)**	**2.8 (2.1-3.6)**
Highest non-school qualification				
Bachelor/post-grad degree	2.2 (0.8-5.7)	0.5 (0.2-1.4)	0.7 (0.3-1.4)	**0.4 (0.3-0.6)**
Diploma	1.2 (0.3-4.8)	0.8 (0.3-2.1)	0.9 (0.4-2.0)	**0.4 (0.2-0.7)**
Certificate	1.1 (0.7-1.8)	1.1 (0.7-1.5)	1.1 (0.8-1.5)	1.0 (0.8-1.3)
No qualifications	1.0	1.0	1.0	1.0
Employment status				
Employed	1.0	1.0	1.0	1.0
Unemployed	**2.0 (1.0-4.0)**	**2.3 (1.3-4.0)**	**2.2 (1.4-3.5)**	**4.6 (2.8-7.5)**
Not in the labour force	1.3 (0.9-2.0)	**2.1 (1.4-3.1)**	**1.9 (1.4-2.6)**	**2.7 (2.3-3.3)**
Housing tenure				
Owner/purchaser	1.1 (0.5-2.7)	**0.4 (0.3-0.7)**	**0.5 (0.4-0.8)**	---
Renter/other tenure	1.0	1.0	1.0	---
Ran out of food and couldn't afford to buy more (last 12 months)				
Yes	**1.8 (1.2-2.7)**	**3.5 (2.4-4.9)**	**2.8 (2.1-3.6)**	**6.3 (4.8-8.2)**
No	1.0	1.0	1.0	1.0
Equivalised household income quintile§				
1 (lowest)	1.8 (0.5-6.7)	2.1 (0.7-6.0)	1.9 (0.8-4.9)	**7.0 (4.5-10.8)**
2	1.5 (0.3-7.1)	1.1 (0.4-3.4)	1.2 (0.5-3.1)	**3.4 (2.1-5.7)**
3	1.0 (0.2-5.6)	1.0 (0.3-3.1)	1.0 (0.4-2.6)	**2.8 (1.8-4.3)**
4	1.0 (0.2-5.7)	0.7 (0.2-2.6)	0.7 (0.2-2.3)	**2.0 (1.2-3.3)**
5 (highest)	1.0	1.0	1.0	1.0
Not known/not stated	1.1 (0.3-4.9)	1.4 (0.5-4.3)	1.3 (0.5-3.3)	**2.7 (1.6-4.4)**
	Trend‖: *p *= 0.07	Trend‖: ***p *= 0.001**	Trend‖: ***p *< 0.001**	Trend‖: ***p *< 0.001**
SEIFA quintile**				
1 (most disadvantaged)	---	2.3 (0.7-7.8)	2.1 (0.6-7.0)	**2.9 (1.9-4.2)**
2	---	1.8 (0.5-6.2)	1.9 (0.6-6.8)	**1.6 (1.1-2.4)**
3	---	1.9 (0.5-6.9)	1.9 (0.6-6.8)	1.4 (0.9-2.1)
4	---	1.1 (0.3-4.3)	1.1 (0.3-4.3)	1.2 (0.8-1.8)
5 (least disadvantaged)	---	1.0	1.0	1.0
	Trend: n/a	Trend: *p *= 0.02	Trend: ***p *= 0.04**	Trend: ***p *< 0.001**

* Source: Weighted data from the National Aboriginal and Torres Strait Islander Health Survey 2004-05 confidentialised unit record file (CURF) [16]. Very high psychological distress is defined as a K5 score of ≥ 15

† OR, odds ratio; CI, confidence interval. All estimates are adjusted for age group (18-24, 25-34, 35-44, 45-54 and 55-64 years). Figures in bold are statistically significant at *p *< 0.05

‡ Includes those who never went to school.

§ Gross weekly equivalised cash income of household, using the OECD scale [17]. Quintiles are based on national figures

‖ Trend analysis excludes those with equivalised household income quintile 'not known/not stated'

** SEIFA, Socioeconomic Index for Areas, Index of Relative Disadvantage. Quintiles are based on national figures

For example, the relative odds of VHPD among those who did not complete Year 10 (compared with those who completed Year 10 or more) was 2.7 (95% CI 1.7-4.3) for Indigenous males in non-remote areas and 3.2 (95% CI 2.3-4.6) for non-Indigenous males; for females, the corresponding figures were 2.2 (95% CI 1.5-3.2) and 2.8 (95% CI 2.1-3.6). There was a significant inverse trend in VHPD with equivalised household income quintile for non-remote Indigenous males and females and non-Indigenous males and females (p ≤ 0.001 in each of the four groups), but the gradient was steeper for non-Indigenous males (OR for a one-unit change in income quintile = 0.47) and females (OR = 0.64) than for their non-remote Indigenous counterparts (0.62 and 0.73, respectively). People who were unemployed were significantly more likely to report VHPD than those who were employed, with relative odds of 5.0 (95% CI 2.4-10.4) for non-remote Indigenous males, 7.2 (95% CI 4.1-12.7) for non-Indigenous males, 2.3 (95% CI 1.3-4.0) for non-remote Indigenous females and 4.6 (2.8-7.5) for non-Indigenous females.

Among Indigenous males and females in remote areas, by contrast, only marital status, main language, and running out of food (and unemployment among females) were significantly associated with VHPD (Tables 3 and 4). Although running out of food was significantly associated with VHPD in all groups examined, the relative odds were smallest among Indigenous people in remote areas (males: OR = 2.3, 95% CI 1.1-4.7; females: OR = 1.8, 95% CI 1.2-2.7) and largest among non-Indigenous people (males: OR = 6.2, 95% CI 4.2-9.1; females: OR = 6.3, 95% CI 4.8-8.2), with Indigenous people in non-remote areas showing intermediate values (males: OR = 4.1, 95% CI 2.5-6.7; females: OR = 3.5, 95% CI 2.4-4.9).

### Impact of VHPD

Among Indigenous respondents with VHPD, 54.7% (95% CI 49.6%-59.9%) indicated that there were days during the last four weeks when they were unable to work or carry out their normal activities, and 31.7% (95% CI 27.0-36.4) said they saw a doctor or other health professional about their feelings in the last 4 weeks. Inability to carry out normal activities was more commonly reported for females (58.6%, 95% CI 52.7-64.4) than for males (47.6%, 95% CI 38.7-56.4) and generally increased with age; the proportions were similar in remote and non-remote areas. Consulting a health professional was also more common among females (34.3%, 95% CI 28.2-40.3) than males (26.9%, 95% CI 18.8-34.9) with VHPD, but this was largely due to the higher proportion among females in non-remote areas (36.5%); females in remote areas (27.5%) were similar to males in remote (26.3%) and non-remote areas (27.0%). Almost one-third (32.1%, 95% CI 27.3-37.0) of Indigenous people with VHPD indicated that physical health problems were the main cause of their feelings all or most of the time, with another 27.1% (95% CI 22.5-31.7) indicating they were the main cause some or a little of the time; the remaining 40.8% (95% CI 35.9-45.6) reported physical health problems as the main cause 'none of the time'. Having physical health problems as the main cause of feelings all or most of the time was more commonly reported in non-remote (34.5%, 95% CI 28.5-40.5) than remote areas (24.2%, 95% CI 17.5-30.9) and increased substantially with age, from 18.3% (95% CI 9.8-26.7) among 18-24 year-olds to 57.8% (95% CI 39.6-76.0) among 55-64 year-olds.

## Discussion

Very high psychological distress was more commonly reported by Indigenous than non-Indigenous Australians in this nationally representative study. The prevalence was associated with most traditional indicators of SES--education, employment, income, home ownership and area-level disadvantage--in both the Indigenous and non-Indigenous populations in non-remote areas, although the relative odds were generally larger in the non-Indigenous population. By contrast, these traditional SES indicators were not significantly associated with VHPD in Indigenous people in remote areas. Other factors, including marital status, main language and food insecurity, were generally significant in all groups, although the relationship between main language and VHPD was not always in the same direction.

The lower prevalence of VHPD among Indigenous people in remote areas, and the lack of association with many socio-demographic variables in this group, suggest that living in an Indigenous-majority environment (such as exists in most remote communities in Australia) may mitigate the risk of psychological distress to some degree. There is evidence from the UK that increased ethnic density is beneficial for minority communities and that this is partially mediated by reduced exposure to racism as well as attenuated impact of such exposure [28,29].

The higher prevalence of VHPD in Indigenous Australians is consistent with other data indicating that Indigenous Australians have a much higher burden of hospitalisation for intentional self-harm as well as mental and behavioural disorders [1]. For Indigenous Australians, 16% of general practitioner visits in 2005-2010 related to mental health problems, compared with 11% for all Australians [30].

Life stressors such as the death of a family member/close friend and alcohol/drug problems or abuse/violent crime among friends or relatives are more common for Indigenous people compared with non-Indigenous people [1] as are higher rates of suicide [31], disability and chronic disease [1]. Indigenous people are also exposed to high levels of racism, trauma and grief [13,32-34].

In addition to its morbid effects, psychological distress is also related to mortality. Using data from the 1997 to 2000 US National Health Interview Survey (NHIS) linked to the US National Death Index through 2002, Pratt found a significantly higher risk of death (during a mean follow-up time of nearly four years) among those with high K6 scores, even after adjusting for a range of variables relating to socio-demographics, health behaviours and physical illness. There was a dose-response relationship between K6 score and risk of mortality [35].

There is evidence that psychological distress is associated with chronic health conditions. For example, in the 2002 NHIS, the prevalence of psychological distress (K6 score ≥ 13) in adults aged 40+ years was higher among those with self-reported congestive heart failure (CHF) (10.0%), myocardial infarction (MI) (6.4%) and coronary heart disease (4.1%) compared with those with no cardiovascular disease (2.8%), with elevated odds of psychological distress among those with MI and CHF remaining after adjustment for a range of socio-demographic and health risk factors [36]. In Australia, in cross-sectional data from almost 78,000 adults aged 18-70 years as part of the Australian Work Outcomes Research Cost-benefit Study, psychological distress (K6 score ≥ 13) was significantly associated with age, sex, marital status, education and income. After adjusting for these factors, psychological distress was associated with a range of health conditions and health risk factors, and the relative odds increased with multiple morbidity [37].

These findings may, in part, explain the increased prevalence of VHPD among Indigenous Australians, who have a greater burden of physical illness and an adverse risk profile. However, in the present study, only about a third of Indigenous participants with VHPD indicated that physical problems were the cause of their feelings most or all of the time. More work is needed to understand Indigenous people's perspectives regarding the causes of VHPD and the role played by physical illness.

For Indigenous males and females as well as for the Indigenous population as a whole, mean scores on the K5 were lower in remote than non-remote areas, and differences between males and females were more pronounced in remote compared to non-remote areas. Other studies have also found regional differences, but the direction of the difference has not always been consistent. In a study of non-metropolitan areas of New South Wales, Kelly et al. found lower mean K10 scores in remote areas than in very remote or inner or outer regional areas; mean scores were similar for males and females [38]. Although non-significant, a similar trend was evident in South Australia with lower mean K10 scores in accessible/moderately accessible areas compared to highly accessible and remote/very remote areas [39]. In the US, Dhingra and colleagues found that, among those in the 2007 Behavioral Risk Factor Surveillance System (BRFSS) (with a landline telephone), urban residents were more likely than rural residents to have mild (K6 7-12 out of 24) or serious (K6 13+) psychological distress after adjustment for socio-demographic characteristics [40].

The higher prevalence of VHPD among Indigenous Australians and the relationship with SES indicators (at least in non-remote areas) are consistent with data on ethnic minority groups in other countries. In New Zealand, K10 scores were significantly associated with sex, age group, education, equivalised household income and area-level deprivation. Maori and Pacific people had higher mean K10 scores than other ethnic groups, even after adjusting for age, sex, educational qualifications and equivalised household income [8]. In the 2002 Canadian Community Health Survey, high K10 scores (> 9 out of 40) were associated with being female, having low education, low income, younger age, and being unmarried; Aboriginal Canadians were more likely to have high psychological distress, but this was only apparent among those of low income [7]. In the 2001-04 NHIS in the US, the prevalence of serious psychological distress in the last 30 days (as indicated by a K6 score ≥ 13 corresponding to K5 ≥ 16) was reported as 3.1% overall. Higher prevalence of serious psychological distress was seen in females, those not married, living in poverty, and who did not complete high school. The relationship between living in poverty and serious psychological distress was observed among Hispanics, Non-Hispanic Whites and Non-Hispanic Blacks alike [6]. In the 2004-08 NHIS, American Indian or Alaska Native (AIAN) adults had a similar prevalence of serious psychological distress (K6 ≥ 13) to that seen in Black and Hispanic adults (3.4% versus 3.4% and 3.5%, respectively), but higher than that in White (2.9%) or Asian (1.4%) adults. However, there were marked differences by sex, with AIAN males having the highest prevalence and AIAN females having the second lowest prevalence among the five ethnic groups examined [41].

These international studies suggest a much lower prevalence of very high psychological distress than that found among Indigenous Australians. However, similar figures were found in a 2003-4 American study, in which 15.4% of 1,202 low-income multi-ethnic workers had a K6 score ≥ 13. This study also found that after adjusting for poverty, psychological distress was significantly associated with workplace abuse and high exposure to racial discrimination [42].

Significant associations among Indigenous Australians have previously been reported for SES and diabetes [43,44], renal disease [45,46] and cardiovascular disease [47] (but not for asthma [48]). The present study indicates that there are significant associations between SES indicators and psychological distress, but these relationships appear to be attenuated, especially in remote areas. This finding is somewhat consistent with data from a study of 963 Indigenous people from a socio-economically disadvantaged coastal region in Australia, in which socio-demographic characteristics were largely non-significant in explaining psychological distress as measured by the K10 [49]. This suggests that risk factors such as racism, with common and/or cross-cutting effects across the socioeconomic spectrum for Indigenous Australians [13,32,33,50], may contribute to psychological distress. In the 2004-05 NATSIHS, those who reported they had been treated badly because they were Aboriginal or Torres Strait Islander were more likely to have high or very high psychological distress (K5 ≥ 12) than those who said they had not been treated badly (39.5% versus 25.0%) [4].

Over half of Indigenous respondents with VHPD in the present study indicated they were not always able to carry out their normal roles/work due to their distress, and about a third had consulted a health professional about their feelings in the last 4 weeks. Such findings accord with international studies demonstrating that psychological distress leads to absence from work both in the short and long term [51,52]. These data serve to further highlight the importance of psychological distress as a health risk factor for Indigenous Australians.

The main strengths of the current study are the use of nationally representative data, comparisons between Indigenous and non-Indigenous populations, and identical socio-demographic measures with comparable scales in the two populations. Although bias is always possible in any survey with less than complete participation, the high response rates in both the NHS and the NATSIHS suggest that any such bias is unlikely to be large. The main limitations relate to the cross-sectional nature of the study and the potential misclassification of VHPD and socio-demographic factors.

Kessler and colleagues have noted that the K6 has minimal bias with respect to age, sex and education [53]. Although bias relating to other factors such as culture or language is possible, the instrument has been used successfully in a wide range of settings. Most notably, the K6 has been validated and used in a diverse group of 14 countries taking part in the World Mental Health survey initiative (Brazil, Bulgaria, Colombia, India, Japan, Lebanon, Mexico, New Zealand, Nigeria, China, Romania, South Africa, Ukraine, and the United States) [53], with additional work in the United States [20,21], Japan [54] and Australia [22]. The K6 has also recently been used in two US Native American populations living on or near reservations and found to be an appropriate screening tool for psychological disorders as well as a good indicator of severity [55].

Although the measure used to assess psychological distress in the NATSIHS was thus based on a widely validated instrument, it has not been widely used before in this population, and one of the questions comprising the K6 was omitted. Previously published data from the 2004-5 NATSIHS suggest that the measure has some validity, based on its association with a range of factors such as positive wellbeing, anger, number of life stressors, mental illness stressor, racial discrimination, and removal from natural family [4].

The data used in this analysis are derived from two different surveys, the NATSIHS and the NHS. Although the ABS planned these two surveys to be run in parallel, with the methodology and question wording matched as closely as possible to allow the data to be compared, some important differences may remain. In particular, NHS participants were administered the full K10, while NATSIHS participants were administered only the K5 (with slightly altered wording), and this may have affected participants' responses. Previous work in the US National Survey on Drug Use and Health has shown variations in K6 scores depending on question order and context [56]. Although it is possible that differences between the NATSIHS and NHS could have affected the results, the extent and direction of any such bias is unclear. We were not able to undertake sensitivity analysis (for example by comparing K5 responses for Indigenous NHS respondents who completed the full K10 and NATSIS respondents who completed only the K5) because the necessary data were not included in the dataset.

Aside from its use in the ABS survey program, we are aware of only one published study using the K5 [57]. In this study of 298 Aboriginal adults aged 15-54 years living in a remote area of Northern Australia, the mean (s.d.) K5 score was 6.58 (2.12). This is substantially lower than the mean K5 score for remote Indigenous males in the NATSIHS (8.27 (0.45)). A study of 184 Indigenous Australians between 2007 to 2009 used a version of the K6 with two additional items (focused on happiness and anger) to validate the Growth and Empowerment Measure (GEM). Findings suggest that, in comparison to the K6 alone, the 'K6 + 2' had slightly higher internal consistency and stronger correlations with the GEM (sub) scales. As such, it may constitute a more valid measure of psychological distress for Indigenous Australians [58].

Psychological distress is only one aspect of mental health and, in turn, mental health only one aspect of social and emotional wellbeing for Indigenous Australians. Previously published data from the 2004 to 2005 NATSIHS indicate that psychological distress does not necessarily correspond to a lack of wellbeing and vice versa. As such, it is important to assess both psychological distress and wellbeing in surveys relating to Indigenous Australians [4].

Information used to determine SES may have been incorrectly reported by (or on behalf of) some participants, and only limited detail was available on the SES indicators examined here. Data on housing tenure was not available in the NATSIHS CURF for the non-Indigenous population. Despite the use of comparable scales, the equivalence of a given level of SES may not be guaranteed across individuals or population groups. For example, the meaning of a certain level of education may vary over time and place, and years of education do not necessarily reflect the quality of education received, nor its social or economic value [59,60].

Similarly, the use of SEIFA quintiles based on the whole population may not adequately capture the socioeconomic position of population subgroups such as Indigenous Australians [61]. No information was available about other potentially important SES measures, such as total household assets or childhood SES. An area-based measure of disadvantage was included, but no other information was available about neighbourhood/area characteristics. Although equivalised household income is intended to adjust for household size and economies of scale, the relatively high mobility of Indigenous people, including movement of individuals across households [62], can make it difficult to assess both Indigenous household income and household size, both of which are required to calculate equivalised income.

Because information on socio-demographic factors and psychological distress were collected at the same time, the temporal relationships between socio-demographic variables and psychological distress are not certain. For example, employment status may change as a result of having psychological distress. This may explain in part the observed relationship between psychological distress and being unemployed or out of the labour force. Similarly, data on physical health problems were collected at the same time as data on psychological distress and were based on self-report. These factors could have influenced the estimate of the proportion of psychological distress explained by physical health problems, although the direction of any such bias is uncertain. Moreover, it is not possible in a national survey to collect information on the full range of the factors that may relate to both psychological distress and SES. Hence, there may be unmeasured confounders which could account for all or part of the findings presented here.

Despite these limitations, the NATSIHS data provide the best available information on psychological distress in Indigenous Australian adults that can be compared directly to the non-Indigenous population.

## Conclusions

This paper demonstrates for the first time in a national survey, the higher absolute levels of VHPD combined with smaller socio-demographic gradients in the Australian Indigenous population. This suggests the relevance of risk factors with cross-cutting effects across the socioeconomic spectrum, such as racism. The lower prevalence of VHPD among Indigenous people in remote areas compared to those in non-remote areas, and the lack of association with many socio-demographic variables in this group, suggest either that the instrument may be less valid for remote-living Indigenous people or that living in an Indigenous-majority environment (such as exists in most remote communities in Australia) may mitigate the risk of psychological distress to some degree.

These findings highlight a clear need for services to meet the needs of Indigenous people with VHPD as well as interventions to address factors which may act as determinants of psychological distress for Indigenous people. The services and interventions that are most appropriate may vary according to factors such as location, language, gender and socioeconomic status. The study also demonstrates the need for further research into the most valid approach to measuring and monitoring levels of psychological distress among Indigenous people as an important aspect of social and emotional wellbeing.

## Abbreviations

ABS: Australian Bureau of Statistics; AIAN: American Indian or Alaska Native; CD: Census collection district; CHF: Congestive heart failure; CURF: Confidentialised unit record file; K5: Kessler Psychological Distress Scale (5-question subset); K6: Kessler Psychological Distress Scale (6-question subset); K10: Kessler Psychological Distress Scale (10 questions); MI: Myocardial infarction; NATSIHS: National Aboriginal and Torres Strait Islander Health Survey; NHIS: National Health Interview Survey; NHS: National Health Survey; OECD: Organisation for Economic Co-operation and Development; RADL: Remote Access Data Laboratory; s.d.: Standard deviation; SES: Socioeconomic status; VHPD: Very high psychological distress.

## Competing interests

The authors declare that they have no competing interests.

## Authors' contributions

JC undertook all data analysis. JC and YCP contributed to conception and design, interpretation of data, drafting the article, and revising it critically for important intellectual content, and both approved the final manuscript.

## Pre-publication history

The pre-publication history for this paper can be accessed here:

http://www.biomedcentral.com/1471-2458/12/95/prepub
